# Induction of targeted, heritable mutations in barley and *Brassica oleracea* using RNA-guided Cas9 nuclease

**DOI:** 10.1186/s13059-015-0826-7

**Published:** 2015-11-30

**Authors:** Tom Lawrenson, Oluwaseyi Shorinola, Nicola Stacey, Chengdao Li, Lars Østergaard, Nicola Patron, Cristobal Uauy, Wendy Harwood

**Affiliations:** John Innes Centre, Norwich Research Park, Colney, NR4 7UH UK; Western Barley Genetics Alliance, Murdoch University, Murdoch, WA6150 Australia; The Sainsbury Laboratory, Norwich Research Park, Colney, NR4 7UH UK

**Keywords:** Genome editing, CRISPR/Cas9, Barley, *Brassica*, *PM19*, *GA4*, Crops, Mutations, Breeding, Off-target

## Abstract

**Background:**

The RNA-guided Cas9 system represents a flexible approach for genome editing in plants. This method can create specific mutations that knock-out or alter target gene function. It provides a valuable tool for plant research and offers opportunities for crop improvement.

**Results:**

We investigate the use and target specificity requirements of RNA-guided Cas9 genome editing in barley (*Hordeum vulgare*) and *Brassica oleracea* by targeting multicopy genes. In barley, we target two copies of *HvPM19* and observe Cas9-induced mutations in the first generation of 23 % and 10 % of the lines, respectively. In *B. oleracea*, targeting of *BolC.GA4.a* leads to Cas9-induced mutations in 10 % of first generation plants screened. In addition, a phenotypic screen identifies T_0_ plants with the expected dwarf phenotype associated with knock-out of the target gene. In both barley and *B. oleracea* stable Cas9-induced mutations are transmitted to T_2_ plants independently of the T-DNA construct. We observe off-target activity in both species, despite the presence of at least one mismatch between the single guide RNA and the non-target gene sequences. In barley, a transgene-free plant has concurrent mutations in the target and non-target copies of *HvPM19*.

**Conclusions:**

We demonstrate the use of RNA-guided Cas9 to generate mutations in target genes of both barley and *B. oleracea* and show stable transmission of these mutations thus establishing the potential for rapid characterisation of gene function in these species. In addition, the off-target effects reported offer both potential difficulties and specific opportunities to target members of multigene families in crops.

**Electronic supplementary material:**

The online version of this article (doi:10.1186/s13059-015-0826-7) contains supplementary material, which is available to authorized users.

## Background

Genetic modification is a key research tool for advancing knowledge of gene function as well as allowing the development of crops with valuable traits. Genetic modification enables the introduction of genes of interest or the reduction in expression of endogenous genes (RNAi approaches) through the insertion of transgenic sequences at random locations within the plant genome. Genetic modification technologies have advanced substantially over the past 30 years, but more recently, a series of exciting developments offer significant opportunities for the analyses of plant genomes, as well as having applications in crop improvement [1]. These approaches, collectively called genome editing, provide the opportunity to make precise changes at specific genomic locations. Genome editing may be used to induce gene insertions, gene replacements, or insertions or deletions that disrupt the function of a specific gene [2]. This latter application, leading to knock-out of target genes, has enormous benefits for research in plants, especially in crops that lack genetic resources such as knock-out libraries.

Genome editing requires a site-directed nuclease to introduce one or more breaks in the DNA at the target locus. The cell’s endogenous DNA repair mechanisms repair these breaks; imperfect repair can produce mutations or deletions in the genes of interest. To generate site-specific breaks, different approaches have employed different combinations of nucleases fused to programmable DNA binding domains including Zinc Finger Nucleases (ZFNs) and Transcription-Activator Like Effector Nucleases (TALENs). More recently, the Cas9 protein associated with Type II Clustered Regulatory Interspaced Short Palindromic Repeats (CRISPR) found in bacteria has been repurposed for genome editing in eukaryotes [3, 4]. The RNA-guided Cas9 system uses a small non-coding RNA, known as the single guide RNA (sgRNA), to direct the Cas9 nuclease to the DNA target of interest. Being small and easy to reprogram, this offers a flexible, easy-to-implement and relatively cheap method for genome editing [5]. The first applications of RNA-guided Cas9 in plants were described in 2013 [6–8] using transient systems. Inheritance of induced mutations in progeny plants was demonstrated for the first time in Arabidopsis by Feng *et al.* [9] and heritable changes have also been shown in rice [10, 11]. In wheat (*Triticum aestivum*), RNA-guided Cas9 has been used to mutate a single homoeologue of the mildew resistance locus *MLO* in stable T_0_ transgenic plants although no information was provided regarding the inheritance of the mutant alleles [12].

Very few studies have described the inheritance of RNA-guided Cas9-induced mutations and questions remain regarding its efficiency, especially in crop plants. In addition, the frequency with which the nuclease induces mutations in unintended targets (known as off-targets) has yet to be extensively investigated across plant species. The aim of this study was therefore to use RNA-guided Cas9 for targeted mutagenesis in both monocotyledonous and dicotyledonous crop species, demonstrating for the first time its application in both barley (*Hordeum vulgare*) and *Brassica oleracea.* In addition, we aimed to assess the efficiency of mutagenesis and test whether off-target effects occurred.

*Arabidopsis GA4* is involved in the gibberellin biosynthesis pathway and *GA4* loss-of-function mutants have dwarf stature and reduced fruit dehiscence [13, 14]. Since plant architecture and seed dispersal are important targets for crop improvement in Brassicas, we tested the effect of mutating *GA4* orthologues in *B. oleracea*. In barley, we chose *HvPM19* as our target. *HvPM19* encodes an ABA-inducible plasma membrane protein [15], which in wheat acts as a positive regulator of grain dormancy [16], an important agronomic trait in cereals.

Here we demonstrate the successful use of RNA-guided Cas9 genome editing to knockout the function of target genes in both barley and *B. oleracea*. We show transmission of the mutation to progeny plants in both species and we demonstrate the segregation of the transgenic locus (encoding the nuclease and sgRNA) from the mutation, resulting in transgene-free plants that contain the desired mutation.

## Results and discussion

### RNA-guided Cas9-induced genome editing in barley

**Fig. 1:**
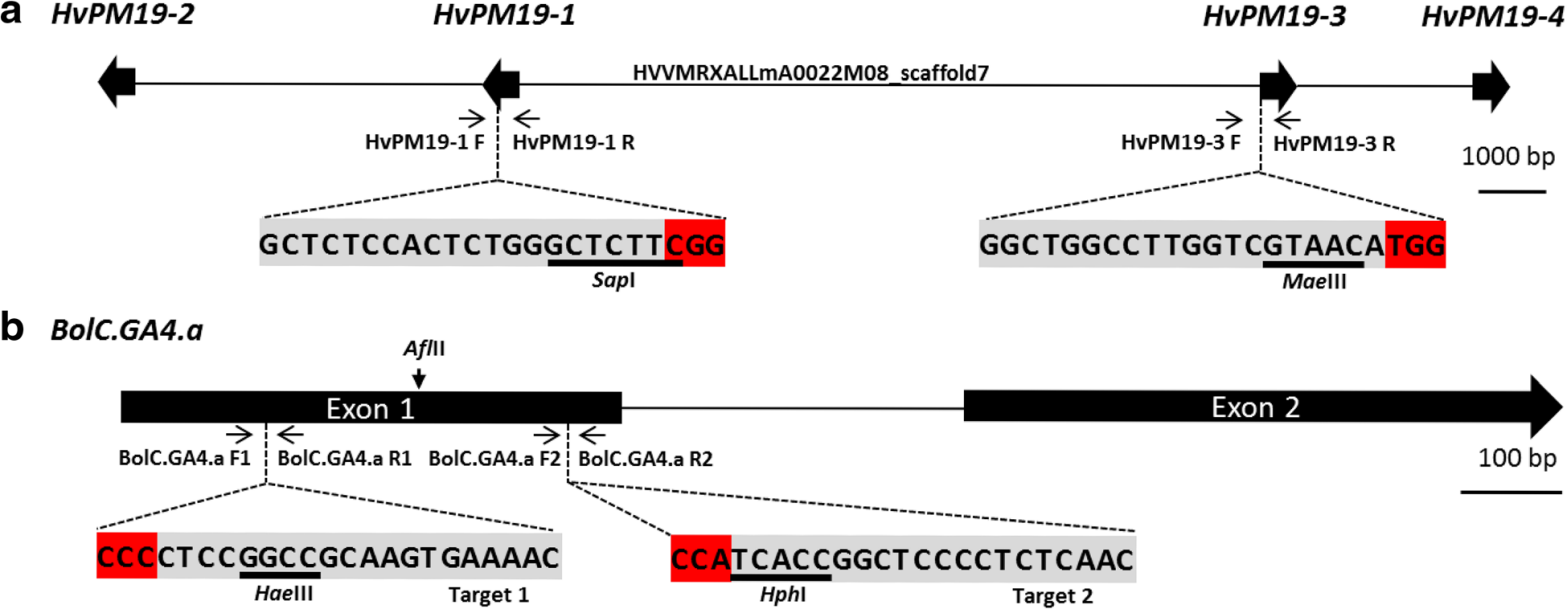
Barley *HvPM19* and *B. oleracea BolC.GA4.a* gene models and target sequences. **a** Morex HVVMRXALLmA0022M08_scaffold7 sequence contains the four barley *HvPM19* gene copies (filled arrows). The target sequences for sgRNAHvPM19-1 and sgRNAHvPM19-3 (grey highlight) are shown below their respective gene models, with the protospacer-adjacent motif (PAM) highlighted in red. Recognition sequences for the restriction endonucleases *Sap*I *and Mae*III are underlined. **b** The *B. oleracea BolC.GA4.a* gene model includes two exons (filled boxes) separated by an intron (represented by a solid line). The *B. oleracea BolC.GA4.a* sequences for sgRNA1BolC.GA4.a (Target 1) and sgRNA2BolC.GA4.a (Target 2) are shown below the target regions in grey highlight with the PAM highlighted in red. Recognition sequences for the restriction endonucleases *Afl*II, *Hae*III and *Hph*I are underlined. Primers for mutant detection are shown in both panels and detailed in Additional file 3

We investigated the use and target specificity requirements of RNA-guided Cas9 genome editing in barley by focusing on a multi-copy gene. We selected *HvPM19,* which is present as four copies within a single barley BAC clone from the cultivar ‘Morex’ (*HvPM19-1* to *HvPM19-4*; Fig. 1a). Relative to *HvPM19-1*, the *HvPM19-2*, *HvPM19-3* and *HvPM19-4* loci have sequence identities of 89.8 %, 89.5 % and 88.6 %, respectively, whereas *HvPM19-3* and *HvPM19-4* have greater sequence identity to *HvPM19-2* (98.4 % and 99.6 %). This suggests that *HvPM19-1* was involved in the more ancestral duplication event and that there was a series of very recent duplication events between *HvPM19-2*, *HvPM19-3* and *HvPM19-4*.

**Fig. 2:**
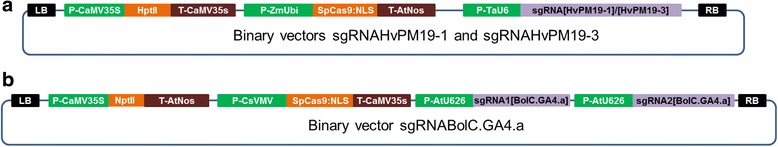
Schematic of binary plasmid vectors delivered to barley and *B. oleracea*. Transcription units were assembled into the binary plasmid backbone pAGM4723 or pAGM8031 using Golden Gate Modular Cloning. **a** The barley constructs, sgRNAHvPM19-1 and sgRNAHvPM19-3 house a hygromycin resistance cassette consisting of the *hygromycin phosphotransferase* coding sequence (*hptII*) driven and terminated by the 35 s promoter (P-CaMV35s) and terminator (T-CaMV35s) from Cauliflower mosaic virus; a Cas9 expression cassette consisting of sequence encoding Cas9 from *Streptococcus pyogenies* with a carboxy-terminal nuclear-localization signal from Simian vacuolating virus 40 (SpCas9:NLS) driven by a ubiquitin promoter from *Zea mays* (*P-ZmUbi*) and terminated by a nopaline synthase terminator from *Agrobacterium tumefaciens* (*T-AtNos*); and single guide RNA (sgRNAHvPM19-1 or sgRNAHvPM19-3) driven by a *Triticum aestivum* U6 promoter (P-TaU6). **b** The *Brassica* construct, sgRNABolC.GA4.a, houses a kanamycin resistance cassette consisting of the *neomycin phosphotransferase* coding sequence (*nptII*) driven and terminated by P-CaMV35S and *T-AtNos*; SpCas9:NLS driven by a constitutive promoter from Cassava Vein Mosaic Virus (P-CsVMV) and a tandem pair of single guide RNAs (sgRNA1BolC.GA4.a and sgRNA2BolC.GA4.a) driven by the U626 promoter from *Arabidopsis* (P-AtU626)

**Fig. 3:**
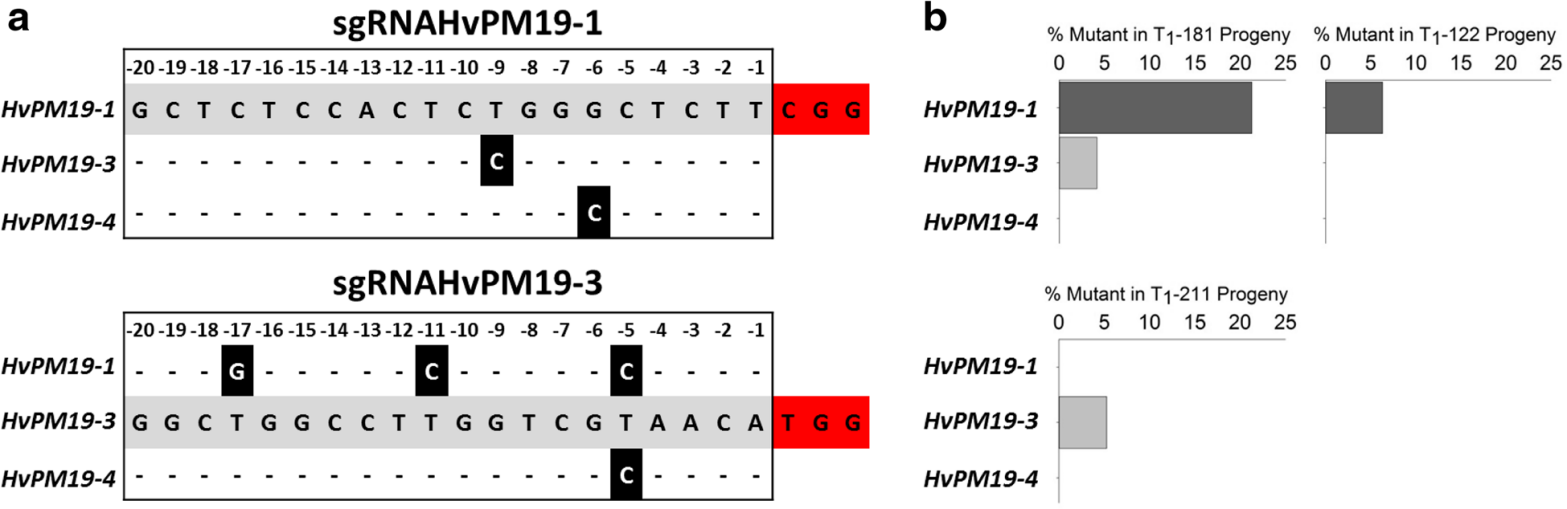
Frequency of on-target and off-target Cas9 activity on the *HvPM19* gene copies at T_1_. **a** Alignment of sgRNAHvPM19-1 and sgRNAHvPM19-3 target sequences (grey highlight) with the corresponding sequences of the other copies of *HvPM19*. Hyphens represent alignment matches while mismatches are shown in black highlight and white font. The PAM is highlighted in red and the numbering of nucleotides is relative to the PAM. **b** Percentage of T_1_ plants with mutations in the corresponding copies of *HvPM19* for sgRNAHvPM19-1 (T_0_-181 and T_0_-122) and sgRNAHvPM19-3 (T_0_-211). Dark and light grey bars represent the percentages for *HvPM19-1* and *HvPM19-3* editing, respectively

We independently targeted two ancestral *HvPM19* gene copies (*HvPM19-1* and *HvPM19-3*) in the spring barley cultivar ‘Golden Promise’ which is amenable to *Agrobacterium*-mediated transformation. We were able to amplify *HvPM19-4* from Golden Promise, but unable to amplify *HvPM19-2* suggesting that this cultivar lacks this copy of *HvPM19*. Two binary constructs, sgRNAHvPM19-1, referred to as pPM19-1 and sgRNAHvPM19-3, referred to as pPM19-3 (Fig. 2a), were designed to independently target *HvPM19-1* and *HvPM19-3*, respectively. The 20 base-pair target sequence in pPM19-1 has a single nucleotide mismatch with each of the corresponding sequences in *HvPM19-3* and *HvPM19-4*, while the target sequence in pPM19-3 has three mismatches with *HvPM19-1* and one mismatch with *HvPM19-4* (Fig. 3a).

The two constructs were independently transformed into immature barley embryos to generate 28 and 20 independent transgenic lines for pPM19-1 and pPM19-3, respectively. T_0_ regenerated plantlets were screened for mutations using a restriction digest/PCR assay. We detected deletions in *HvPM19-1* in three out of 13 pPM19-1 T_0_ lines screened (T_0_-181, T_0_-122 and T_0_-191). Similarly, out of the 10 pPM19-3 T_0_ plantlets screened, one line (T_0_-211) showed an insertion in *HvPM19-3*. Therefore, the frequency of Cas9-induced mutations in the first generation was 23 % for pPM19-1 and 10 % for pPM19-3. These mutation frequencies are comparable to those reported in stable T_0_ transformants from other monocotyledonous species such as wheat [12], rice (*Oryza sativa*; reviewed in [17]) and sorghum (*Sorghum bicolor*; [18]).

As is characteristic of Cas9-induced mutations [3, 4, 19], all the insertions or deletions (in-dels) detected were at the 3' end of the target region, 3 or 4 bp upstream of the Protospacer Adjacent Motif (PAM; Additional file 1). In the T_0_ plants, we detected in-dels only after enriching for the mutation by the restriction digest/PCR assay presumably because only a small proportion of the cells had been mutated at the time of sampling. To further characterise the events, we measured T-DNA copy number in the T_0_ lines and found that T_0_-181, T_0_-191 and T_0_-211 each contained a single copy of the T-DNA whereas T_0_-122 contained two copies.

### On-target and off-target activity of RNA-guided Cas9 in T_1_ transgenic barley plants

Next, we examined the T_1_ progenies of T_0_-181, T_0_-122, T_0_-191 and T_0_-211. Twenty out of 93 T_1_ progenies of T_0_-181 contained in-dels in the target *HvPM19-1* sequence as determined by Sanger sequencing. Of these, two were homozygous and 18 were heterozygous mutants determined by the presence of double peaks in the sequencing chromatogram beginning from the site of the in-del. For T_0_-122, only six out of 95 T_1_ progenies had in-dels in the target *HvPM19-1* sequence, with all being heterozygous mutations. This represents mutation frequencies of 22 % in T_0_-181 and 6 % in T_0_-122 progenies. For line T_0_-211, which showed Cas9 activity for *HvPM19-3*, we detected four mutant plants out of 76 T_1_ progenies tested, all of which had heterozygous mutations (mutation frequency of 5 %). Line T_0_-191 showed mutations in seven out of 90 T_1_ plants, but was not analysed further. As in the T_0_ generation, all the in-dels were observed to occur in the 3–4 bp adjacent to the PAM. All the T_1_ plants with Cas9-induced mutations retained their corresponding T-DNA construct, while there was segregation in the non-mutated T_1_ plants. This indicated that the mutations could still be the product of sgRNA/Cas9 expression in somatic cells rather than due to germline inheritance.

To assess the specificity of the T-DNA constructs, we sequenced *HvPM19-3* and *HvPM19-4* in the progenies of T_0_-181 and T_0_-122 (designed to target *HvPM19-1*). We found no off-target activity in the T_1_ progenies of T_0_-122; whereas three T_0_-181 progeny from 72 tested (4.2 %) had off-target activity on *HvPM19-3* (Fig. 3b). By contrast, we observed no off-target activity on *HvPM19-1* and *HvPM19-4* in the 73 T_1_ progenies of T_0_-211 that contained the T-DNA designed to target *HvPM19-3*.

### Cas9-induced mutations are stably transmitted to T_2_ barley plants independently of the T-DNA construct

The mutation in the target gene theoretically should segregate independently of the T-DNA that encodes the nuclease and sgRNA. We observed complete co-segregation of the Cas9-induced mutations with the T-DNA construct in the T_1_ transgenic lines. We therefore tested the T_2_ progenies of several T_1_ lines to determine if the mutations could be stably inherited and segregate independently from the T-DNA construct. We screened for the presence of the T-DNA through PCR and qPCR assays and determined the mutation status in the T_2_ progenies of T_1_-181, T_1_-122 and T_1_-211 lines (T_1_ mutant lines originating from the corresponding T_0_ lines; Table 1). The T-DNA segregated in the progeny of some, but not all, of these T_1_ lines. Segregation of pPM19-1 was detected in 11 out of 19 T_1_-181 lines, whereas pPM19-1 segregated in the progeny of four out of six T_1_-122 lines. However, only one out of three T_1_-211 lines tested showed segregation of the pPM19-3 construct. A 3:1 presence:absence ratio was confirmed using a χ^2^ test in all progenies in which the T-DNA segregated (*P* >0.44 or higher).

**Table 1:** Summary of transgenerational RNA-guided Cas9 activity and segregation in barley

T-DNA Construct	T_0_ line	T_1_ mutation type	T_1_ line	Number of T_2_ plants screened for T-DNA	Plants without T-DNA	Plants without T-DNA and with (homozygous/heterozygous) mutations^a^
pPM19-1	T_0_-181	Homozygous	T_1_-181_B5	4	0	-
			T_1_-181_E1	12	5	5/0
		Heterozygous	T_1_-181_A11	12	3	1/1
			T_1_-181_B1	8	2	0/0
			T_1_-181_B8	11	0	-
			T_1_-181_C1	9	0	-
			T_1_-181_C12	1	0	-
			T_1_-181_C3	12	0	-
			T_1_-181_C4	12	1	0/0
			T_1_-181_C9	11	1	0/1
			T_1_-181_D11	12	4	1/1
			T_1_-181_D2	12	3	2/0
			T_1_-181_D9	2	0	-
			T_1_-181_E4	12	0	-
			T_1_-181_G4	11	3	0/1
			T_1_-181_G5	12	1	0/0
			T_1_-181_H2	12	3	1/1
			T_1_-181_H5	12	0	-
			T_1_-181_H9	12	4	0/0
	T_0_-122	Heterozygous	T_1_-122_B11	12	4	2/1
			T_1_-122_C6	12	1	0/1
			T_1_-122_F12	12	4	0/3
			T_1_-122_H2	12	0	-
			T_1_-122_H4	12	3	0/0
			T_1_-122_H9	12	0	-
pPM19-3	T_0_-211	Heterozygous	T_1_-211_B11	12	3	0/0
			T_1_-211_D10	12	0	-
			T_1_-211_G4	7	0	-

^a^Hyphens (-) indicate that all plants had presence of the T-DNA construct, and thus were not tested

We next sequenced *HvPM19-1* and *HvPM19-3* from all 45 T_2_ plants that had not inherited the T-DNA. In these plants, we detected mutations in *HvPM19-1* in 15 T_2_ progenies originating from seven independent T_1_-181 lines and seven T_2_ progenies originating from three independent T_1_-122 lines (Table 1). A single progeny of T_1_-181_H2 showed an off-target mutation in *HvPM19-3* in addition to the on-target *HvPM19-1* mutation. Interestingly, T_1_-181_H2 is one of the three T_0_-181 lines that showed off-target activity of pPM19-1 in the T_1_ generation. For pPM19-3, we did not detect mutations in *HvPM19-3* (and *HvPM19-1*) in the absence of the T-DNA in the progenies of any of the T_1_-211 lines.

**Fig. 4:**
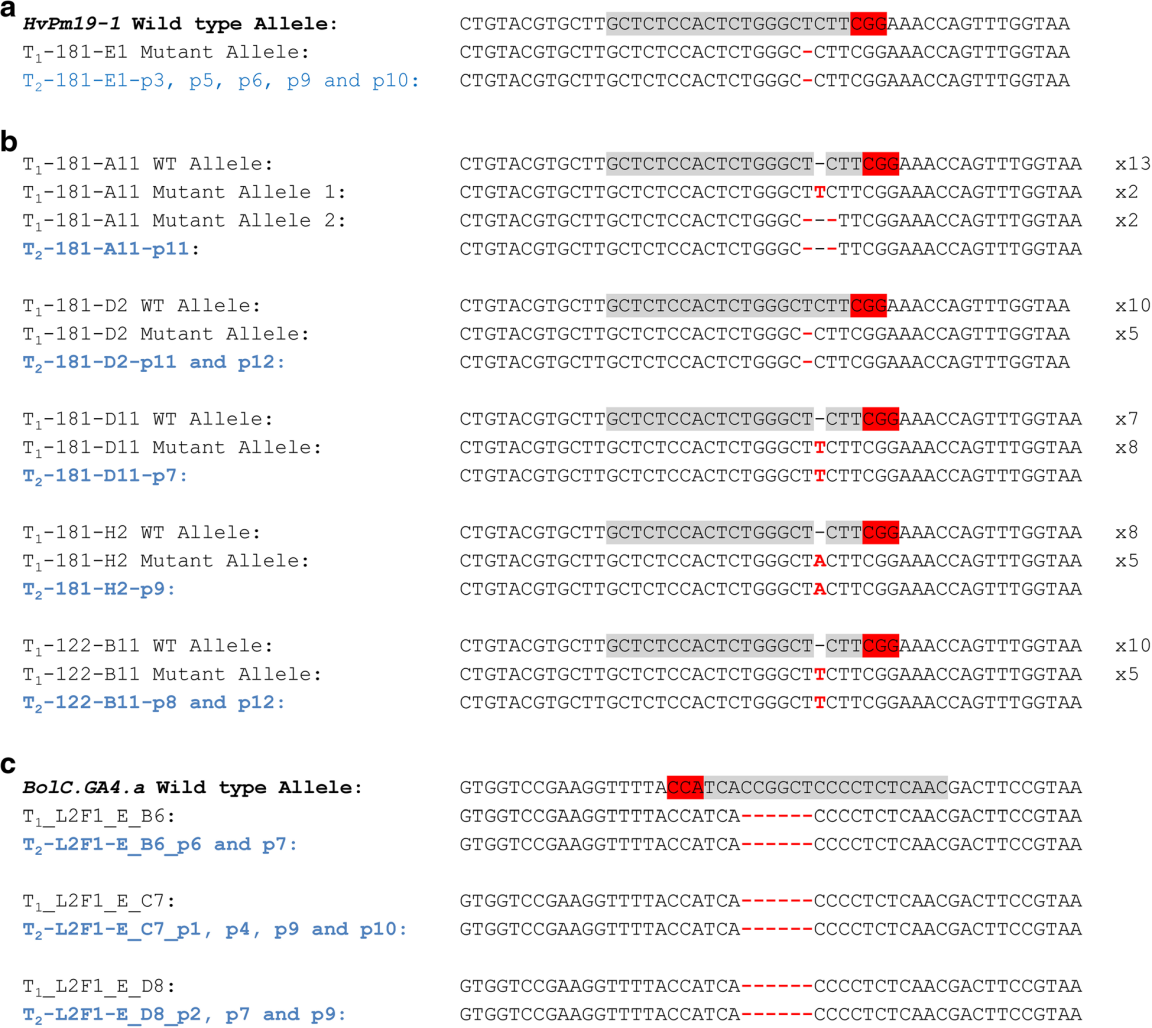
Germline transmission of Cas9 induced mutations from T_1_ to T_2_ plants in barley and *B. oleracea* in the absence of the T-DNA construct. **a** Sequence alignment of T_1_-181_E1 and five homozygous T_2_ progeny with homozygous 1-bp deletion in *HvPM19_1*. **b** Sequence alignment from representative clones of T_1_ heterozygote mutants and direct Sanger sequencing of their T_2_ progeny with homozygous mutations in the absence of the T-DNA. The numbers of clones supporting T_1_ mutant alleles are indicated on the right. **c** Sequence alignments of *BolC.GA4.a* Target 2 in homozygous T_1_ and T-DNA free T_2_ plants. Across panels the target sequences for sgRNAHvPM19-1 and sgRNABolC.GA4.a (grey) and PAM (red) are highlighted and Cas9 induced insertions and deletions are indicated by red font or red hyphens, respectively. Names of homozygous T_2_ plants that lack the presence of the T-DNA construct are indicated in blue; individual homozygous plants with the same allele are shown on the same row and are labelled with a ‘p’ prefix

We found that the mutations detected in the T_2_ progenies matched those observed in the corresponding T_1_ parent in all cases examined. For instance, the homozygous 1-bp deletion observed in line T_1_-181_E1 was also present in all its T_2_ progenies that segregated away from the T-DNA construct (Table 1; Fig. 4a). Likewise, a range of mutations found in heterozygous T_1_ plants were also identified in homozygous T_2_ individuals in the absence of the T-DNA construct (Fig. 4b). Taken together, the T_1_ and T_2_ sequence data from six lines, originating from two independent T_0_ events (T_0_-181 and T_0_-122), provide strong evidence of stable germline transmission of Cas9-induced mutations in barley in the absence of the T-DNA.

The ability to develop transgene-free and stable germline mutations is of considerable interest in crop species given the current regulatory framework for deployment of transgenic crops in the field. Although regulation of edited crops is still being debated [20], crops free of transgenes may not be subject to existing regulations on genetic modification. Here, we demonstrate that in several instances there was stable germline-transmitted inheritance of Cas9-induced mutations in barley from the T_1_ to the T_2_ generation in the absence of the T-DNA construct. This supports previous studies in plants (Arabidopsis, tomato, tobacco and rice) that have documented transgene-free inheritance of Cas9-induced mutations in the T_1_ and T_2_ generations. No description of germline inheritance has been previously reported for *Triticeae* [12]. We also identified a single plant with an off-target mutation in the T_2_ generation in the absence of the T-DNA construct. This plant had mutations in both *HvPM19-1* and *HvPM19-3*, suggesting that tandemly duplicated genes can be knocked-out with a single sgRNA, although we have yet to establish if these mutations are in *cis* or on homologous chromosomes. Previous work in rice had identified off-target mutations only in the T_1_ generation and in the presence of the sgRNA/Cas9 construct [21].

### RNA-guided Cas9 induced genome editing in *Brassica oleracea*

RNA-guided Cas9-induced genome editing was performed in *B. oleracea* DH1012 [22] by targeting *BolC.GA4.a* (*Bol038154*) located on chromosome 5*.* This gene is an orthologue of *Arabidopsis GA4* which encodes AtGA3OX1, the last enzyme in the biosynthesis of bioactive gibberellins. In *Arabidopsis*, *ga4* loss-of-function mutants show a semi-dwarf phenotype [23] and this gene is required for efficient seed dispersal as it promotes specification of the dehiscence zone in siliques [13]. *BolC.GA4.a* has a paralog on chromosome 8, designated *BolC.GA4.b* (*Bol031570*), which shares 90 % DNA sequence identity. To generate Cas9 induced mutations in *BolC.GA4.a,* we designed a binary construct containing two sgRNAs (sgRNA1BolC.GA4.a and sgRNA2BolC.GA4.a) that target separate regions (Target 1 and Target 2, respectively) in the first exon of *BolC.GA4.a* (Figs. 1b and 2b).

**Fig. 5:**
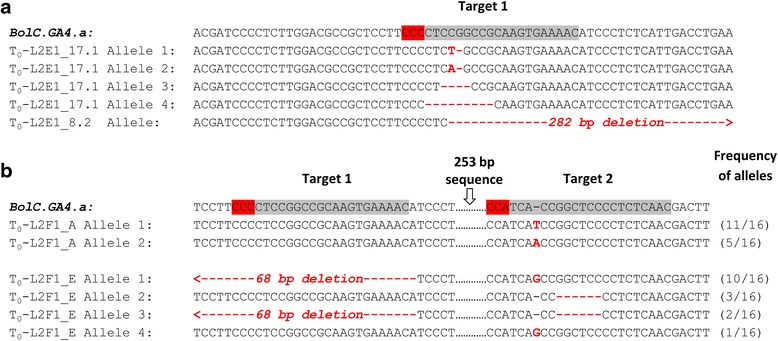
Mutant alleles detected in T_0_ *B. oleracea*. Alignment of wild-type and mutant sequences surrounding the target sequences (grey highlight) and PAM (red highlight) in mutants identified by restriction digest/PCR screen (**a**) and by phenotypic screen (**b**). Insertions and deletions are indicated by red font or red hyphens, respectively. For large deletions, red arrows indicate the direction of the deletions. For each line in panel b (L2F1_A and L2F1_E), 16 clones were examined and the frequencies of each mutant allele (represented as clones with mutant allele/total number of clones examined) are indicated at the right side of the panel

Eighty independent transgenic lines were generated by *Agrobacterium*-mediated transformation, and 20 of these T_0_ plantlets were screened by the restriction digest/PCR assay to detect mutations in the target sequences. We identified in-dels at the target sites in *BolC.GA4.a* in two out of 20 T_0_ lines (L2F1_8.2 and L2E1_17.1). Mutations in L2E1_17.1 were confirmed by TA cloning and Sanger sequencing of the PCR products (Fig. 5a). Line L2F1_8.2 showed a 282-bp deletion that corresponds to re-joining of the DNA at exactly 3 bp from the PAM in both target regions. As in barley, the detection of the mutations required an enrichment of the target by restriction digest prior to PCR.

**Fig. 6:**
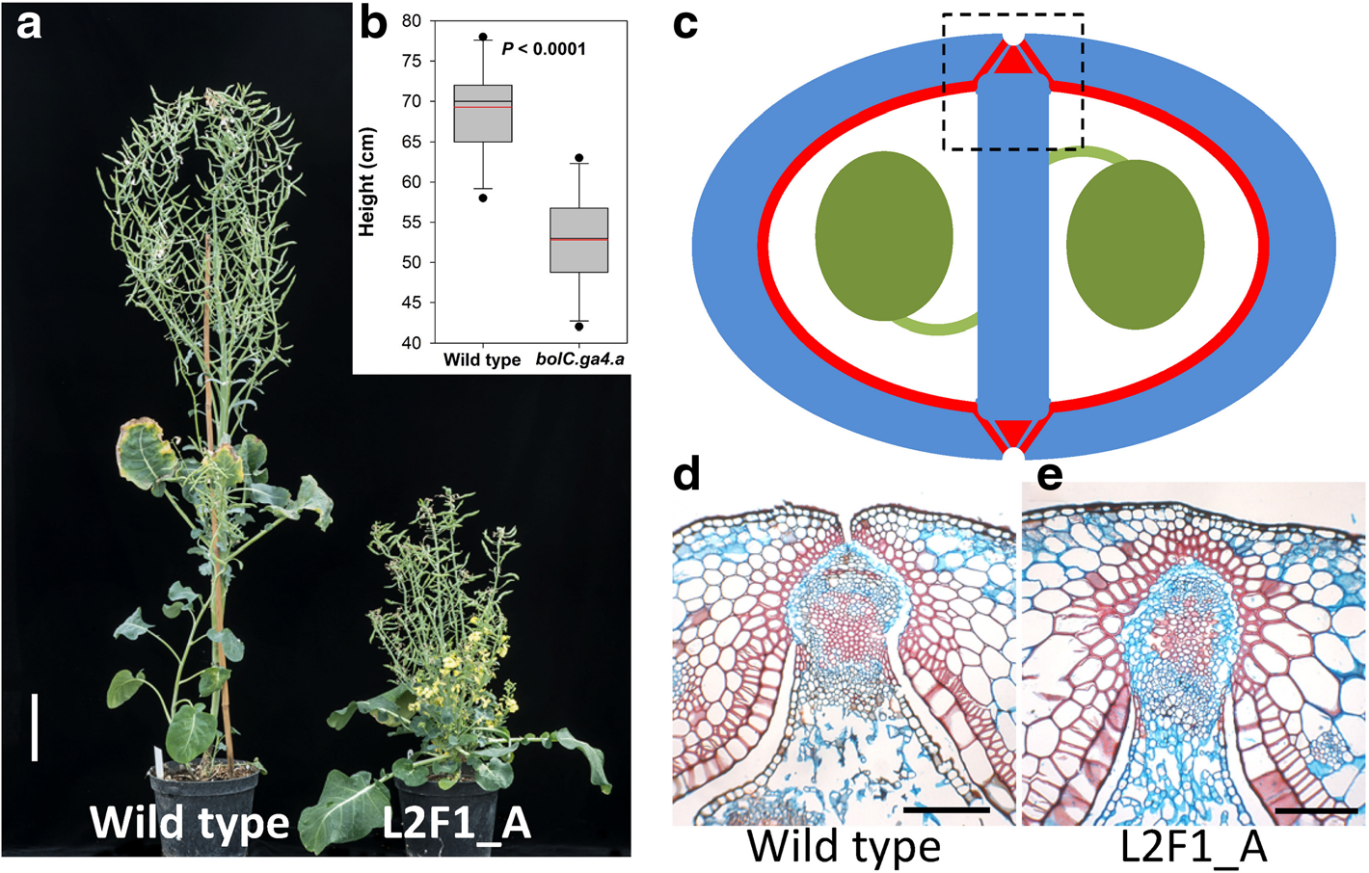
Mutations in *BolC.GA4.a* result in dwarf stature and affect the pod valve margin. **a** Wild-type *B. oleracea* DH1012 (left) and L2F1_A with a mutation in *BolC.GA4.a* showing a severe dwarf phenotype. Scale bar 10 cm. **b** Height of homozygous T_1_ plants with wild type (n = 11) or *bolC.ga4.a* mutant (n = 16) alleles. **c** Schematic cross section of *B. oleracea* pod with replum/valve margin region indicated by dashed square. Lignified tissue is indicated in red, unlignified cells are indicated in blue, and developing seeds are in green. **d, e** Cross-section of replum valve margin region of *B. oleracea* wild-type pod (**d**) and L2F1_A mutant pod (**e**); scale bars 200 μm

We also hypothesised that plants with homozygous mutations in *BolC.GA4.a* would show a dwarf phenotype similar to that observed in Arabidopsis *ga4* mutants. Therefore, we performed a phenotypic screen of the 80 T_0_*B. oleracea* plants. All 80 T_0_ lines were grown to maturity, and at flowering two lines not previously characterised by the restriction digest/PCR assay were observed to be dwarf in stature (L2F1_A and L2F1_E; Fig. 6a). The *BolC.GA4.a* sequences from both dwarf plants were found to contain a series of mutant alleles in Target 1 and Target 2, in two independent leaf samples from each plant (Fig. 5b, Additional file 2). In addition, the mutation was restricted to *BolC.GA4.a*, as we were unable to detect any mutation in *BolC.GA4.b*. The identification of T_0_ plants with a visible knockout phenotype has also been reported in rice, tomato, and Arabidopsis [8, 10, 24].

The 80 T_0_*B. oleracea* plants described above originated from the same transformation experiment, but differed in their culture period. A first batch of 41 T_0_ shoots was isolated four weeks after *Agrobacterium* inoculation, whereas a second batch of 39 T_0_ shoots was isolated 7 weeks after inoculation. Both dwarf lines were derived from the 7-week batch, supporting a recent report in rice [25] that obtained an increased proportion and variety of mutated cells by extending the culture period of rice calli by 4 weeks. Across different target genes, Mikami *et al.* [25] found a 3.7-fold increase in mutation frequencies between rice calli cultured for 1 month compared to 2 months. They hypothesize that this is due to a greater chance of inducing novel mutations in non-mutated cells [25]. Our results are consistent with this hypothesis which also implies that shorter selection periods during culture of calli could reduce the number of off-target mutations.

### Cas9-induced mutations are stably transmitted to T_2_*B. oleracea* plants independently of the T-DNA construct

**Fig. 7:**
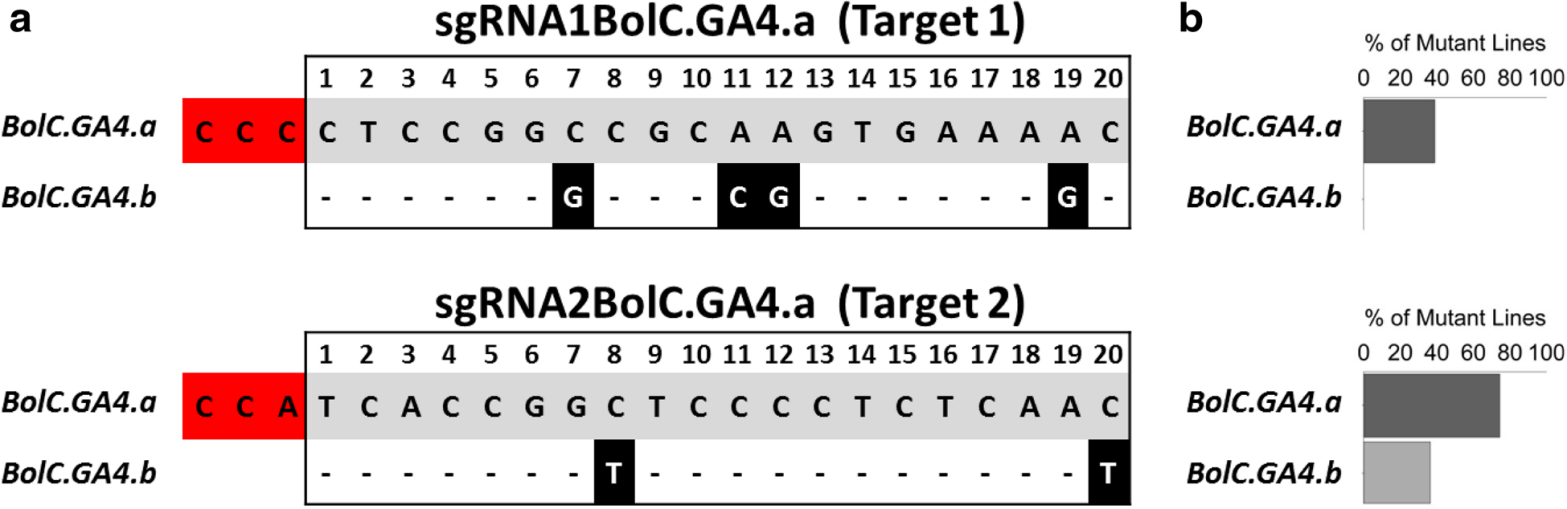
Frequency of on- and off-target Cas9 activity in L2F1_8.2 T_1_ *Brassica* plants. **a** The alignment of sgRNA1BolC.GA4.a and sgRNA2BolC.GA4.a target sequences in *BolC.GA4.a* with their corresponding sequences in *BolC.GA4.b*. Hyphens represent alignment matches while mismatches are shown in black highlight and white font. The PAM is highlighted in red and numbering of nucleotides is relative to the PAM. **b** Percentage of the T_1_ plants with mutations in *BolC.GA4.a* and *BolC.GA4.b.* Dark and light grey bars represent the percentages of *BolC.GA4.a* and *BolC.GA4.b* editing, respectively. N = 90 plants

To examine the mutation frequency of the target locus *BolC.GA4.a*, the T_1_ progenies of lines L2F1_8.2 and L2E1_17.1 were screened for Cas9-induced mutations in Target 1 and 2 by PCR amplification of *BolC.GA4.a* followed by direct sequencing. Using the sequencing chromatograms it was possible to identify homozygous and heterozygous mutations. We detected mutations in the T_1_ progenies of L2F1_8.2, but not in L2E1_17.1. Heterozygous in-dels were observed in 68 of 90 L2F1_8.2 T_1_ progenies; however, no homozygous mutations were identified. Of these 68 T_1_ plants, 35 had mutations in Target 1, whereas Target 2 was mutated in 67 lines, suggesting a higher efficiency of the Target 2 sgRNA (Fig. 7b). None of the 90 T_1_ progenies inherited the complete 282-bp deletion between the two *BolC.GA4.a* target regions that was observed in the T_0_ generation.

The sgRNA targets were also sequenced in the T_1_ progenies of lines L2F1_A and L2F1_E that showed a dwarf phenotype. T_1_ plants from each of L2F1_A and L2F1_E were screened and found to carry a range of either homozygous or heterozygous mutations across the target regions in *BolC.GA4.a* (Additional file 2). Of the 39 plants screened, 20 had either homozygous mutations or a combination of two mutant alleles previously identified in the T_0_ plants; all of these plants displayed the dwarf phenotype at maturity (Fig. 6b). In the remaining 19 heterozygous lines we also identified the same mutations as in the T_0_ plants, including the large 68-bp deletion across Target 1 (Fig. 5b; Additional file 2). These results are consistent with stable transmission of Cas9-induced mutant alleles in *B. oleracea*.

To determine germline inheritance, we screened for the presence of the T-DNA construct in 12 individual T_2_ plants derived from eight homozygous mutant T_1_ lines (96 plants in total). Nine T_2_ plants which lacked the T-DNA construct were recovered (Fig. 4c). These plants all carried the same 6-bp deletion in Target 2, and wild-type allele in Target 1, as in the parental T_1_ plants (L2F1_E_B6, L2F1_E_C7, and L2F1_E_D8). The fact that the mutations in T_1_ plants were stably transmitted to the T_2_ generation in the absence of the T-DNA construct supports the germline inheritance of the Cas9- induced mutations in *B. oleracea*.

### Off-target activity of RNA-guided Cas9 in T_1_ transgenic *B. oleracea* plants

Sequencing of *BolC.GA4.b* in the T_1_ progenies of T_0_-L2F1_8.2 revealed off-target activity in 32 out of 88 plants (36.4 %; Fig. 7b). This was restricted to Target 2 where the sgRNA contained two mismatches with *BolC.GA4.b* (Fig. 7a). No mutations were observed across Target 1 where the sgRNA contained four mismatches in the target region compared to *BolC.GA4.b*. In T_0_ lines with the dwarf phenotype (T_0_ lines L2F1_A and L2F1_E), we detected no off-target activity in *BolC.GA4.b*, indicating that the dwarf phenotype was due to mutations in *BolC.GA4.a* only.

In this study, we show that a single sgRNA (sgRNA2BolC.GA4.a) can simultaneously target two copies of *GA4* despite the presence of a mismatch between the sgRNA and the *BolC.GA4.b* off-target sequence 8 bp from the PAM (Fig. 7a). This observation mirrors our results in barley, in which off-target activity was detected in *HvPM19-3* due to the sgRNA in pPM19-1, designed to target *HvPM19-1* and which has a mismatch 9 bp from the PAM (Fig. 3). This off-target activity was detected only in the progeny of the *B. oleracea* and barley lines with high on-target mutation frequencies. Given these results and the idea that on-target mutations may precede off-target mutations [26], it is tempting to speculate that higher on-target Cas9 activity positively correlates with higher off-target mutation frequencies.

These results differ from reports in wheat, where a single mismatch 3 bp from the PAM between *MLO* homoeologues limited off-target activity, although on-target mutation frequencies were relatively low (5.6 %; [12]). Previous studies found that a single mismatch within the 12 bp adjacent to the PAM could confer specificity in humans and other systems [3, 27]. However, others [28, 29] have shown that up to two mismatches, as well as small insertions and deletions, are tolerated within this sequence. Taken together, these results suggest that additional work is needed to decipher the key design rules and experimental parameters relating to on- and off-target mutations using the Cas9 system.

The presence of off-target activity can be considered a negative feature of the Cas9 system when specificity is sought. Several approaches have been suggested for the reduction of off-target activity. These include using truncated sgRNAs [30], a pair of Cas9 nickase mutants directed to opposing strands that require a pair of correctly positioned 20 bp DNA targets to produce a DSB [31, 32], and also the fusion of catalytically dead Cas9 (dCas9) to homodomains of a FokI nuclease dimer that will also only produce a DSB when both targets are in correct proximity [33, 34]. However, off-target activity can also be beneficial for targeting gene families [26] or closely related sequences. Our results suggest that a single sgRNA can simultaneously target multiple gene copies facilitating gene functional analysis by overcoming possible redundancy between the closely related sequences [35]. Importantly, we identified an individual transgene-free barley plant that had concurrent heterozygous mutations in the target (*HvPM19-1*) and off-target (*HvPM19-3*) genes. Many crop species are polyploid (for example, wheat, potato), have undergone recent whole-genome duplication events (for example, Brassica, maize; [36]), or have a high number of tandemly duplicated genes [37], such as the *HvPM19* locus investigated in this study. Therefore, the potential to generate progeny with mutations limited to on-target sites, as well as progeny with both on- and off-target mutations, makes the RNA-guided Cas9 system especially relevant for functional analyses in crops.

### Mutations in *BolC.GA4.a* affect the pod valve margin

Tissue patterning in the fruits of Arabidopsis and members of the Brassica genus is highly similar reflecting their close evolutionary relationship [38]. Seed dispersal in these species depends on formation of valve margin cells along the valve and replum borders that mediate fruit opening [39]. Since valve margins from Arabidopsis *ga4* mutants fail to mediate efficient seed dispersal [13], we tested if the *B. oleracea* Cas9 lines presented here suffered from similar defects. Cross-sections stained with a combination of Safranin O and Alcian Blue revealed that in comparison to wild-type fruits, fruits from L2F1_A failed to pattern the valve margin region properly, such that valve cells replaced the valve margin cells in this line (Fig. 6c-e). As a result, these fruits would disperse their seeds less efficiently than wild type. Although less severe, this phenotype resembled the phenotype observed when another regulator of valve margin formation, *BolC.IND.a*, was downregulated by RNAi [38]. These data therefore demonstrate that the *BolC.GA4.a* function is conserved between *B. oleracea* and *Arabidopsis* and likely regulated in a similar fashion. They also demonstrate the potential for the use of RNA-guided Cas9 to target important traits in *Brassica* crops based on knowledge of gene function from model plants.

## Conclusions

In this study, we demonstrate the use of RNA-guided Cas9 to induce targeted mutations in two crop species, *B. oleracea* and barley, and report stable transmission of the mutations across generations. We show that knock-out phenotypes can be recovered as early as the primary T_0_ generation, exemplifying the use of this technology for rapid analyses of gene function. We produced transgene-free barley and *B. oleracea* plants with stably-inherited mutations in the target gene, supporting the potential for downstream biotechnological applications. Both species showed off-target activity, despite the presence of at least one mismatch between the sgRNA and the paralogous gene. This led to the identification of a single barley plant with concurrent mutations in the target and off-target gene in the absence of the T-DNA construct. Our results suggest that experimental parameters relating to on- and off-target mutations need to be carefully considered and monitored, and that a single sgRNA has the potential to generate progeny with simultaneous knock-out mutations in paralogous genes. Given that crop genomes commonly contain multiple closely related sequences, the features described herein make RNA-guided Cas9 especially relevant for functional analyses in these species.

## Materials and methods

### Target locus selection and sgRNA design

Gene sequences for *B. oleracea BolC.GA4.a* (*Bol038154*) and barley *HvPm19* (AF218627.1; [15]) were obtained from The Brassica Database [40] and the International Barley Sequencing Consortium [41] databases. For barley, sequence of the BAC clone HVVMRXALLmA0022M08 from the cultivar ‘Morex’ was kindly provided pre-publication by Dr Nils Stein (IPK). This BAC was annotated and four copies of *HvPm19* were identified (*HvPM19-1* to *HvPM19-4*). Target sequences that conformed to G(N)_20_GG were identified on sense and antisense strands in the coding sequence for *BolC.GA4.a* and for *HvPM19-1* and *HvPM19-3* and potential off-target sequences were detected via BLAST searches [40, 41]. Potential targets were also evaluated for the presence of non-CpG sensitive restriction site sequences predicted to be disrupted by Cas9 induced in-dels, which also had to be unique within a PCR amplicon. Final target sequences were chosen to be as specific as possible to the intended target sequence (that is, keeping the number of mismatches to off-target sequences high), close to the start codon, and to include an appropriate restriction site (Fig. 1). These targets were checked by PCR and Sanger sequencing (Additional file 3) in the varieties to be transformed (spring barley cultivar ‘Golden Promise’ and *Brassica oleracea* DH1012) to ensure that no polymorphisms existed between the sgRNA and the target G(N)_20_GG sequences. Single sgRNAs were used for barley *HvPM19-1* and *HvPM19-3*, whereas two independent sgRNAs were targeted to the first exon of *Brassica BolC.GA4.a*. Barley ‘Golden Promise’ sequences for the three *HvPM19* genes were deposited in GenBank (accession numbers KT336449-KT336451).

### Construct assembly

The binary plasmid vector constructs were assembled using Golden Gate Modular Cloning (MoClo) [42]. We used Level 0 parts from the Golden Gate MoClo Plant Parts Kit (Addgene kit # 1000000047) and plasmids from Golden Gate MoClo Plant Toolkit (Addgene kit # 1000000044) described in Engler *et al*. [43]. Level 1 transcriptional units were assembled from Level 0 parts and these were subsequently assembled to make the plasmids vectors shown in Fig. 2. A detailed protocol for the assembly of binary vectors with multiple sgRNAs using the Golden Gate MoClo ToolKit and the identity of all plasmids used are given in Additional file 4. Annotated sequences of the plasmids made in this study are provided in Additional file 5 and are available at the non-profit plasmid depository AddGene (https://www.addgene.org/browse/article/14759/).

### Plant transformation and screening of transgenic material

Barley (*cv.* ‘Golden Promise’) was transformed by *Agrobacterium tumefaciens-*mediated transformation of immature embryos as described by Harwood [44]. *Brassica oleracea* (DH1012) was transformed by *Agrobacterium tumefaciens* infection of 4-day-old cotyledonary petioles according to Hundleby and Irwin [45].

Primary transgenic T_0_ materials were screened using a modified restriction enzyme site loss method [46]. Briefly, for single sgRNA targets, genomic DNA was digested prior to PCR with a CpG-insensitive enzyme to remove wild-type template and thus favour the PCR amplification of mutant DNA where the restriction site had been lost. For *Brassica*, where a pair of sgRNAs was used, an additional screen was implemented; a CpG-insensitive restriction enzyme (*Afl*II) was used prior to PCR to enrich for mutant DNA where the fragment between the two guides had been removed. PCR amplification across the region thus led to shorter PCR products than expected from a wild-type individual.

DNA was extracted according to Edwards *et al.* [47] from rooted shoots of less than 10 cm in height and quantified using a Nanodrop 8000 (Thermo Scientific). Genomic DNA (100 ng) was digested overnight with 20 units of the appropriate restriction enzyme shown in Fig. 1 (*Sap*I, *Hae*III, *Hph*I, *Afl*II (NEB); *Mae*III (Roche)) and then purified using a Qiagen QIAquick Gel Extraction Kit (final elution with 25 uL of water). Purified digested DNA (5 μL) was used as PCR template to amplify across the target regions using gene-specific primers (Additional file 3). PCR products were confirmed by agarose gel electrophoresis, purified using the QIAquick Gel Extraction Kit, and Sanger sequenced (Eurofins MWG) to confirm the presence of in-dels. Where the amplicon was too short for direct sequencing, the PCR product was first cloned using the pGEMT-Easy kit (Promega) according to the manufacturer’s instructions and then sequenced with M13 universal primers.

The detection of mutations in T_1_ and T_2_ transgenic lines was performed though Sanger sequencing of PCR amplicons produced using genomic DNA template that was not digested prior to PCR (Additional file 3). Sequences were compared to wild type to detect the presence of homozygous in-dels. Chromatograms were also examined to identify overlapping traces in the region surrounding the PAM, indicative of the presence of mutations. The presence of the T-DNA construct was assessed in progenies of active lines by PCR amplification of the *nptII* CDS in *Brassica* and *hptII* CDS in barley (Additional file 3).

### Phenotyping of *B. oleracea* transgenic lines

The 80 primary T_0_ transgenic lines and corresponding controls were grown in a controlled environment room with 16 h light (high-pressure sodium lamps with an average bench reading of 200 μmol/m^2^/s) at 12 °C and 8 h dark at 12 °C and constant 65–75 % humidity. Plant height was measured at final maturity. Seed pods at developmental stage 17 [48] were collected from dwarf line L2F1_A and the wild-type DH1012 control. Pods were fixed for 16 h in FAA solution (3.7 % formaldehyde, 5 % acetic acid, 50 % ethanol) and subsequently dehydrated through an ethanol series consisting of 50 %, 60 %, 70 %, 80 %, 90 %, 95 %, and 100 % ethanol for 30 min each at room temperature. The tissues were cleared with Histoclear (National Diagnostics,) and embedded in paraffin wax. Transverse sections 8 μm thick were cut using an RM 2055 rotary microtome (Leica) and mounted on Polysine™ slides (VWR International).The wax was removed using Histoclear and sections stained using an Alcian Blue/Safranin-O solution (0.05 % Alcian Blue and 0.01 % Safranin-O in 0.1 M acetate buffer (pH5.0)) as described by Østergaard *et al*. [49]. Sections were examined by light microscopy using a Zeiss Axioplan microscope and images captured using a Leica DFC 320 camera with Leica Application Suite software.

### T-DNA copy number and presence/absence determination in transgenic barley

Quantitative real-time PCR was used to determine copy number (T_0_) and presence/absence (T_2_) of the T-DNA in transgenic barley and *B. oleracea* lines. The reaction compared the Cq values of an *HptII* (Fig. 2a) amplicon to a single-copy barley gene *CO2* (*Constans-like*, AF490469) amplicon and the Cq values of an *NptII* amplicon to a single-copy *B. oleracea* gene *GL2-like* (*Bol021421*) within a single multiplexed assay (Additional file 3). The reactions used Thermo ABGene Absolute QPCR Rox Mix (Cat number AB1139) with the probes and primers at a final concentration of 200 nM (*HptII* and *NptII*) and 100nM (*CO2* and *GL2*). The assay contained 5 μL DNA solution, and was optimised for final DNA concentrations of 1 to 10 ng/μL (5 to 50 ng DNA in the assay). PCRs were carried out in a Bio-Rad CFX96 machine (C1000 Touch). The detectors used were FAM-TAMRA and VIC-TAMRA for barley and HEX-BHQ1 and FAM-BHQ1 for *B. oleracea*. The PCR cycling conditions were 95 °C for 15 min (enzyme activation), 40 cycles of 95 °C for 15 s, and 60 °C for 60 s. Each sample was analysed twice and for presence/absence determinations, two independent DNA extractions of the T_2_ transgenic plants were used. Cq values were determined using the accompanying CFX96 software (version 3.1), with Cq determination set to regression mode. Values obtained were used to calculate T-DNA copy number according to published methods [50].

## Additional files

Additional file 1:
**Chromatogram traces of T**_**0**_ **-181 and T**_**0**_ **-191 plants.** (JPG 439 kb) Additional file 2:
**Analysis of** ***BolC.GA4.a*** **sequences in L2F1_A and L2F1_E T**_**0**_ **and T**_**1**_ **plants.** (DOCX 25 kb) Additional file 3:
**Primers and probes used in this study.** (XLSX 12 kb) Additional file 4:
**Detailed methods for assembly of binary vectors with multiple sgRNAs using the Golden Gate MoCloToolKit.** (DOCX 28 kb) Additional file 5:
**Genbank files (.gb) of the 14 plasmids submitted to AddGene.** (ZIP 41 kb)
