# Supplementary figures and images for: Induction of targeted, heritable mutations in barley and Brassica oleracea using RNA-guided Cas9 nuclease
Source: Genome Biol. 2015 Nov 30;16:258. doi: 10.1186/s13059-015-0826-7 (PMC4663725; doi:10.1186/s13059-015-0826-7)

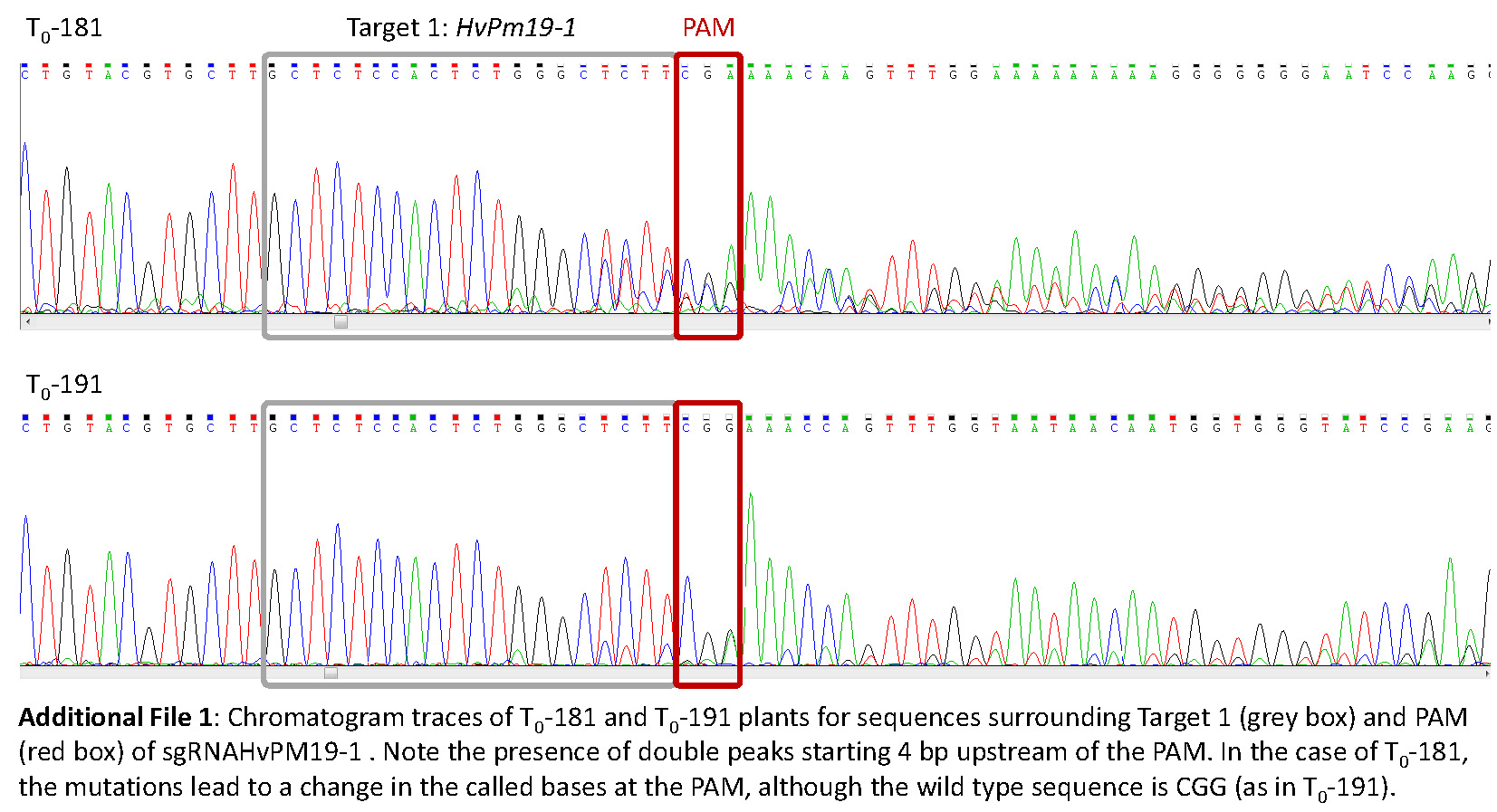

Supplement: Additional file 1: — Chromatogram traces of T 0 -181 and T 0 -191 plants. (JPG 439 kb) [file 13059_2015_826_MOESM1_ESM.jpg]
