# Supplementary material for: Induction of targeted, heritable mutations in barley and Brassica oleracea using RNA-guided Cas9 nuclease
Source: Genome Biol. 2015 Nov 30;16:258. doi: 10.1186/s13059-015-0826-7 (PMC4663725; doi:10.1186/s13059-015-0826-7)
Supplement: Additional file 2: — Analysis of BolC.GA4.a sequences in L2F1_A and L2F1_E T 0 and T 1 plants. (DOCX 25 kb) [file 13059_2015_826_MOESM2_ESM.docx]

**Additional File 2: Analysis of *BolC.GA4.a* sequences in L2F1_A and L2F1_E T_0_ and T_1_ plants**

***Sequencing of T_0_ plants:***

We sequenced the original DNA stocks from the T_0_ dwarf lines L2F1_A and L2F1_E across *BolC.GA4.a* Targets 1 and 2. The PCR product was sub-cloned and 16 clones sequenced to determine the alleles present in the T_0_ lines.

For L2F1_A we identified two alleles: one carried a wild-type sequence across Target 1 and a +T insertion in Target 2 (11 of 16 clones), whereas the second allele was also wild-type across Target 1 but carried a +A insertion in Target 2 (five of 16 clones).

| T_0_ L2F1_A | Target 1 allele | Target 2 allele |
| --- | --- | --- |
| 11 clones | Wild type | +T insertion |
| 5 clones | Wild type | +A insertion |

For L2F1_E we identified four alleles made up of the combinations of two sequences at each of the two targets. For Target 1 we identified the wild-type allele and a 68-bp deletion spanning the region, whereas for Target 2 we identified a +G insertion and a 6-bp deletion. These four alleles were found in the following frequency:

| T_0_ L2F1_E | Target 1 allele | Target 2 allele |
| --- | --- | --- |
| 10 clones | 68-bp deletion | +G insertion |
| 3 clones | Wild type | 6-bp deletion |
| 2 clones | 68-bp deletion | 6-bp deletion |
| 1 clone | Wild type | +G insertion |

***Inheritance in T_1_ plants:***

We PCR amplified and direct sequenced Target 1 and Target 2 for *BolC.GA4.a* in 39 T_1_ progeny from the two dwarf lines L2F1_A and L2F1_E and found a series of alleles in homozygous and heterozygous state.

For L2F1_A we identified the same alleles as seen in the T_1_ progeny, as well as the combination of the two alleles (+T and +A insertions in heterozygous state).

| T_1_ L2F1_A | Target 1 allele | Target 2 allele |
| --- | --- | --- |
| 8 plants | Wild type | +T insertion |
| 6 plants | Wild type | +A insertion |
| 4 plants | Wild type | +A insertion/+T insertion |

For L2F1_E we identified the same two alleles which were most frequent in the 16 T_0_ clones: (a) the 68-bp deletion at Target 1 linked with the +G insertion in Target 2 and (b) the wild-type Target 1 sequence linked with the 6-bp deletion in Target 2.

| T_0_ L2F1_E | Target 1 allele | Target 2 allele |
| --- | --- | --- |
| 15 plants | 68-bp deletion | +G insertion |
| 6 plants | Wild type | 6-bp deletion |

*Cloning and sequencing of homozygous T_1_ plants*:

To further confirm these results we examined eight of the T_1_ plants which we determined to be homozygous from this analysis. We PCR amplified across Target 1 and Target 2, sub-cloned the PCR product and Sanger sequenced 12 independent clones. In all cases we confirmed all 12 clones to carry the expected allele in homozygous state.

A graphical summary of the T0 and T1 *BolC.GA4.a* alleles is shown below:

**L2F1_A**

**L2F1_E**
