# Supplementary material for: Induction of targeted, heritable mutations in barley and Brassica oleracea using RNA-guided Cas9 nuclease
Source: Genome Biol. 2015 Nov 30;16:258. doi: 10.1186/s13059-015-0826-7 (PMC4663725; doi:10.1186/s13059-015-0826-7)
Supplement: Additional file 4: — Detailed methods for assembly of binary vectors with multiple sgRNAs using the Golden Gate MoCloToolKit. (DOCX 28 kb) [file 13059_2015_826_MOESM4_ESM.docx]

**Additional File 4:** **Detailed methodologies for assembly of binary vectors with multiple sgRNAs using the Golden Gate MoClo ToolKit**

Gene expression cassettes encoding Cas9 and sgRNAs are introduced into the plant genome along with a plant selectable marker cassette.

The Golden Gate MoClo Assembly standard described in Engler et al (2014) dictates that transcriptional units are assembled from Level 0 parts into in Level 1 Acceptor plasmids. Subsequently assembled Level 1 constructs are assembled into level 2 or M acceptors. Below we describe the assembly of Level 1 transcriptional units for plant selectable marker cassettes as well as Cas9 and sgRNA expression cassettes.

**A. Assembly of plant selectable marker cassettes**

Level 1 selectable marker cassettes were assembled from the following Level 0 components and Level 1 acceptors in a one-step digestion ligation reaction. New Level 0 parts were made according to the methods described in Engler *et al.* (2014). Reactions were conducted as described in section D below. Except where noted (with as asterisk) plasmids were obtained from the Golden Gate MoClo Plant Parts Kit (Addgene kit # 1000000047) and the Golden Gate MoClo Plant Toolkit (Addgene kit # 1000000044) described in Engler *et al.* (2014).

| **Barley** | | **Brassica** | |
| --- | --- | --- | --- |
| **Plasmid** | **Insert** | **Plasmid** | **Insert** |
| pICH51266 (AddGene# 50267) | Promoter (1.3 kb), 35s (Cauliflower Mosaic Virus) + 5'UTR omega (Tobacco Mosaic Virus) | pICH51288 (AddGene) #50269 | Promoter (double), 35s (Cauliflower Mosaic Virus) + 5'UTR, omega (Tobacco Mosaic Virus) |
| pICSL80036* (AddGene #68259) | Coding sequence, hygromycin phophotransferase II (*Escherichia coli*) | pICSL80037* (AddGene #68260) | Coding sequence, neomycin phophotransferase II (*Escherichia coli*) |
| pICH41414 (AddGene #50337) | 3' untranslated region and terminator, 35s (Cauliflower Mosaic Virus) | pICH41421 (Addgene #50339) | 3' untranslated region and terminator, nopaline synthase (*Agrobacterium tumefaciens*) |
| pICH47732 (AddGene #48000) | Binary Backbone; Level 1 Position 1 acceptor | pICH47732 (AddGene #48000) | Binary Vector Backbone; Level 1 Position 1 acceptor |

| *New to this manuscript - the annotated Genbank (.gb) file of these plasmid sequence are also available in Addition File 5. |
| --- |

**B. Assembly of Cas9 expression cassettes**

Level 1 Cas9 expression cassettes were assembled from the following Level 0 components and Level 1 acceptors in a one-step digestion ligation reaction. New Level 0 parts were made according to the methods described in Engler *et al.* (2014). Reactions were conducted as described in section D below. Except where noted (with as asterisk) plasmids were obtained from the Golden Gate MoClo Plant Parts Kit (Addgene kit # 1000000047) and the Golden Gate MoClo Plant Toolkit (Addgene kit # 1000000044) described in Engler *et al.* (2014).

| **Barley** | | **Brassica** | |
| --- | --- | --- | --- |
| **Plasmid** | **Insert** | **Plasmid** | **Insert** |
| pICSL12009* (AddGene #68257) | Promoter and 5' untranslated region, Ubiquitin (Zea mays) | pICSL12006 (Addgene #50270) | Promoter and 5' untranslated region, Cassava Vein Mosaic Virus |
| pICH41308 (Addgene#49770) | Cas9 (Streptococcus pyogenes) codon optimised for expression in humans** | pICH41308 (Addgene#49770) | Cas9 (Streptococcus pyogenes) codon optimised for expression in humans** |
| pICH41421 (Addgene #50339) | 3' untranslated region and terminator, nopaline synthase (*Agrobacterium tumefaciens*) | pICH41414 (AddGene #50337) | 3' untranslated region and terminator, 35s (Cauliflower Mosaic Virus) |
| pICH47742 (AddGene #47742) | Binary Vector Backbone; Level 1 Position 2 acceptor | pICH47742 (AddGene #47742) | Binary Vector Backbone; Level 1 Position 2 acceptor |

*New to this manuscript - the annotated Genbank (.gb) files of these plasmid sequence are also available in Addition File 5.

**Kindly received from Sophien Kamoun (Nekrasov V, Staskawicz B, Weigel D, Jones JD, Kamoun S: Targeted mutagenesis in the model plant *Nicotiana benthamiana* using Cas9 RNA-guided endonuclease. Nat Biotechnol 2013;31:691–3).

**C. Assembly of sgRNA expression cassettes**

Target sequences were selected from confirmed genomic sequences conforming to
5' **G**NNNN NNNNN NNNNN NNNNN **NGG** 3'

The target sequence was integrated into a double stranded DNA molecule ready for assembly with a U6 promoter in a Level 1 Golden Gate reaction using a 5' tailed oligonucleotide tailed primer to amplify the sgRNA from an existing sgRNA containing plasmid (Addgene#46966 a gift from Sophien Kamoun).

For barley the forward primers were as follows:

tgtggtctca CTTG NNNN NNNNN NNNNN NNNNN ***gttttagagctagaaatagcaag***
(The *Bsa*I recognition site is in blue; the four base pair overhang produced by digestion with *Bsa*I is in underlined capitals – this fuses to the last four base pairs of the AtU6-26 promoter in plasmid pICSL90002; the 20 bp target sequence is in red; the portion of the oligonucleotide that anneals to the sgRNA template in in bold italics)

For brassica the forward primers were as follows:

tgtggtctca ATTG NNNN NNNNN NNNNN NNNNN ***gttttagagctagaaatagcaag***
(The *Bsa*I recognition site is in blue; the four base pair overhang produced by digestion with *Bsa*I is in underlined capitals – this fuses to the last four base pairs of the AtU6-26 promoter in plasmid pICSL90002; the 20 bp target sequence is in red; the portion of the oligonucleotide that anneals to the sgRNA template in in bold italics)

The following reverse primer was used for both species:

tgtggtctca AGCG ***taatgccaactttgtac***
(The *Bsa*I recognition site is in blue; the four base pair overhang produced by digestion with *Bsa*I is in underlined capitals – this fused to the Level 1 acceptor plasmid; the portion of the oligonucleotide that anneals to the sgRNA template in in bold italics

This was done using Phusion DNA polymerase (NEB) following the manufacturer’s instructions. This produced the following PCR amplicon:

tgtggtctcaA/CTTGNNNNNNNNNNNNNNNNNNNGTTTTAGAGCTAGAAATAGCAAGTTAAAATAAGGCTAGTCCGTTATCAACTTGAAAAAGTGGCACCGAGTCGGTGCTTTTTTTCTAGACCCAGCTTTCTTGTACAAAGTTGGCATTACGCTtgagaccaca

Amplicons were verified by agarose gel electrophoresis from which they were subsequently cut and purified using a Qiaquick gel extraction kit (Qiagen). After quantification an appropriate amount of DNA was used in a Level 1 assembly reaction with the following plasmids:

| **Barley** | | **Brassica** | |
| --- | --- | --- | --- |
| **Plasmid** | **Insert** | **Plasmid** | **Insert** |
| pICSL9003* (AddGene #68262) | Promoter, U6 (*Triticum aestivum*) | pICSL90002* (AddGene #68261) | Promoter, U6-26 (*Arabidopsis thaliana*) |
| n/a | PCR amplicon from sgRNA template (amplified from Addgene#46966 with primers described above) | n/a | PCR amplicons from sgRNA PCR template (amplified from Addgene#46966 with primers described above) |
| pICH47751 (AddGene #48002) | Level 1, position 3 acceptor | pICH47751 (AddGene #48002) | Level 1, position 3 acceptor |
|  |  | pICH47761 (AddGene #48003) | Level 1, position 4 acceptor |

*New to this manuscript - the annotated Genbank (.gb) files of these plasmid sequence are also available in Addition File 5.

**D. Protocol for assembly of Level 1 transcriptional units**

Level 1 assembly reactions contained 100–200 ng of the Level 1 acceptor plasmid as well as Level 0 plasmids or sgRNA amplicon such that inserts to be included in the acceptor backbone were at a 2:1 molar ratio to the acceptor. The reaction mix included 10 units of *Bsa*I (NEB), 2 μL of 10X BSA, 400 units of T4 DNA ligase (NEB) and 2 μL of T4 ligase buffer (provided with T4 ligase). Reaction volumes were made up to 20 μL using sterile distilled water. Reactions were incubated in a thermocycler as follows: 26 cycles of 37 °C for 3 min/16 °C for 4 min followed by 50 °C for 5 min and finally 80 °C for 5 min.

A total of 2 μL of each reaction was immediately transformed into chemically competent *E. coli* cells (Invitrogen). Cells were spread on LB agar plates containing 100 mg/L carbenicillin (Melford), 25 mg/L IPTG (Melford) and 40 mg/L Xgal (Melford). White colonies were selected and the fidelity of the clone confirmed by restriction digest analysis and Sanger sequencing.

**E. Assembly of Level M binary vectors with multiple sgRNAs**

The level 1 constructs assembled above were combined into Level M acceptor plasmids to make the final binary vectors delivered to plants (Fig. 2). The following Level 1 constructs, end-linkers and Level M acceptors were used. Except where noted (with as asterisk) plasmids were obtained from the Golden Gate MoClo Plant Toolkit (Addgene kit # 1000000044) described in Engler *et al.* (2014).

| **Barley** | | **Brassica** | |
| --- | --- | --- | --- |
| **Plasmid** | **Insert** | **Plasmid** | **Insert** |
| pICSL11059* (AddGene #68263) | Barley plant selection cassette (see above) | pICSL11055* (AddGene #68252) | Brassica plant selection cassette |
| pICSL11056* (AddGene #68258) | Barley Cas9 cassette | pICSL11060* (AddGene #68264) | Brassica Cas9 cassette |
| pICSL11057* (AddGene #68253) | Barley sgRNA cassette targeting PM19_1 | pICSL11061* (AddGene #68255) | Brassica sgRNA cassette 1 targeting BolC.GA4a |
| pICSL11058* (AddGene #68254) | Barley sgRNA cassette targeting PM19_3 | pICSL11062* (AddGene #68256) | Brassica sgRNA cassette 2 targeting BolC.GA4a |
| pICH50892 (AddGene #48046) | Position 3 end linker | pICH50900 (AddGene #48047) | Position 4 end linker |
| pAGM8031 (AddGene #48037) | Binary Vector Backbone; Level M acceptor | pAGM8031  (Addgene #48037) | Binary Vector Backbone; Level M acceptor |

*New to this manuscript - The annotated Genbank (.gb) files of these plasmid sequence are also available in Additional File 5.

Level M assembly reactions contained 100–200 ng of the Level M acceptor plasmid as well as Level 1 plasmids such that inserts to be included in the acceptor backbone were at a 2:1 molar ratio to the acceptor. The reaction mix included 20 units of *Bpi*I ThermoFisher), 2 μL of 10X BSA, 400 units of T4 DNA ligase (NEB) and 2 μL of T4 ligase buffer (provided with T4 ligase). Reaction volumes were made up to 20 μL using sterile distilled water. Reactions were incubated in a thermocycler as follows: 26 cycles of 37 °C for 3 min/16 °C for 4 min followed by 50 °C for 5 min and finally 80 °C for 5 min.

A total of 2 μL of each reaction was immediately transformed into chemically competent *E. coli* cells (Invitrogen). Cells were spread on LB agar plates containing 100 mg/L Spectinomycin (Sigma), 25 mg/L IPTG (Melford) and 40 mg/L Xgal (Melford). White colonies were selected and the fidelity of the clone confirmed by restriction digest analysis and Sanger sequencing.
